# Osteochondrosis in the central and third tarsal bones of young horses

**DOI:** 10.1177/03009858231185108

**Published:** 2023-07-11

**Authors:** Kristin Olstad, Stina Ekman, Sigriður Björnsdóttir, Cathrine T. Fjordbakk, Kerstin Hansson, Sigurdur F. Sigurdsson, Charles J. Ley

**Affiliations:** 1 Norwegian University of Life Sciences, Ås, Norway; 2 Swedish University of Agricultural Sciences, Uppsala, Sweden; 3 Agricultural University of Iceland, Hvanneyri, Iceland

**Keywords:** central tarsal bone, endochondral ossification, horse, intramembranous ossification, osteochondrosis, third tarsal bone

## Abstract

Recently, the central and third tarsal bones of 23 equine fetuses and foals were examined using micro-computed tomography. Radiological changes, including incomplete ossification and focal ossification defects interpreted as osteochondrosis, were detected in 16 of 23 cases. The geometry of the osteochondrosis defects suggested they were the result of vascular failure, but this requires histological confirmation. The study aim was to examine central and third tarsal bones from the 16 cases and to describe the tissues present, cartilage canals, and lesions, including suspected osteochondrosis lesions. Cases included 9 males and 7 females from 0 to 150 days of age, comprising 11 Icelandic horses, 2 standardbred horses, 2 warmblood riding horses, and 1 coldblooded trotting horse. Until 4 days of age, all aspects of the bones were covered by growth cartilage, but from 105 days, the dorsal and plantar aspects were covered by fibrous tissue undergoing intramembranous ossification. Cartilage canal vessels gradually decreased but were present in most cases up to 122 days and were absent in the next available case at 150 days. Radiological osteochondrosis defects were confirmed in histological sections from 3 cases and consisted of necrotic vessels surrounded by ischemic chondronecrosis (articular osteochondrosis) and areas of retained, morphologically viable hypertrophic chondrocytes (physeal osteochondrosis). The central and third tarsal bones formed by both endochondral and intramembranous ossification. The blood supply to the growth cartilage of the central and third tarsal bones regressed between 122 and 150 days of age. Radiological osteochondrosis defects represented vascular failure, with chondrocyte necrosis and retention, or a combination of articular and physeal osteochondrosis.

Developmental orthopedic diseases are a group of clinical entities that are frequently diagnosed in young horses.^30^ The most common of them is osteochondrosis, defined as a focal disturbance in endochondral ossification.^18^ Endochondral ossification occurs within growth cartilage found in 2 locations at either end of long bones: the metaphyseal growth plate, or physis, located between the primary diaphyseal and secondary epiphyseal centers of ossification, and the articular-epiphyseal cartilage complex that surrounds the secondary ossification center. Osteochondrosis arises as a consequence of failure of the blood supply to growth cartilage,^6,19^ which tends to be organized as anatomical end arteries that run inside cartilage canals.^24^ Vessels are believed to fail because they traverse junctions between biomechanically different tissues, such as the junction between growth cartilage and subchondral bone^24,34^ and the junction between perichondrium and growth cartilage at sites where ligaments also attach.^10^ In epiphyseal growth cartilage, vascular failure results in ischemic necrosis of chondrocytes outside diffusion distance from alternative sources, known as osteochondrosis latens.^6,19,24,34^ With time, the ossification front advances to surround the area of necrosis and this causes a delay in endochondral ossification, or osteochondrosis manifesta.^6,19,24,34^ Articular (ischemic-necrotic) osteochondrosis lesions can resolve or progress to osteochondrosis dissecans (OCD) fragments^8,19^ or pseudochondral or true subchondral bone cysts.^21^ In physeal growth cartilage, vascular failure results in retention of morphologically viable hypertrophic chondrocytes.^22^ The physis is destined to be entirely replaced with bone, thus most lesions resolve,^22^ but physeal (retained-hypertrophic) osteochondrosis has been associated with angular limb deformity manifesting some months later.^27^

Cuboidal bones are subject to the same osteochondrosis, OCD, and cysts as long bones,^31^ but they also suffer additional developmental orthopedic diseases, such as incomplete ossification,^9,14^ collapse,^9,16^ wedging,^16,29^ and distal tarsal osteoarthritis known as juvenile arthritis or bone spavin.^4,12,31^ Watrous et al^31^ examined the distal tarsal bones of foals from 90 days of age and described lesions containing “augmentation of the hypertrophied layer,” chondrocyte degeneration, and necrosis, which appear to have similarities with both articular^6,24^ and physeal^22^ osteochondrosis. Watrous et al^31^ did not mention whether lesions were centered on necrotic vessels, but recently the blood supply to the central (CTB) and third (TIII) tarsal bones of 23 equine fetuses and foals up to 150 days of age was studied using arterial barium perfusion and micro-computed tomography (CT).^28^ Generalized changes were detected in 3 cases and were compatible with incomplete ossification in two of them.^28^ Focal defects in the ossification front were detected in 14 cases, including 1 case with incomplete ossification, and were interpreted as radiological osteochondrosis.^28^ When compared with perfused vessels, the geometry of the radiological osteochondrosis defects matched the configuration of vertical, transverse, or circumferential vessels, supporting that they were the result of vascular failure,^28^ but this requires histological confirmation. The aim of this study was to examine CTBs and TIIIs from these 16 cases and to describe the tissues present, cartilage canals, and lesions, including suspected osteochondrosis lesions.

## Materials and Methods

### Cases

The material originated from the previous micro-CT study of Sigurdsson et al^28^ in which the CTB and TIII of 23 equine fetuses and foals up to 150 days of age were examined in relation to distal tarsal osteoarthritis. The fetuses and foals died or were euthanized at stud farms in Iceland or at the University Animal Hospital of the Norwegian University of Life Sciences. In Iceland, all owners gave verbal consent and, in Norway, all owners gave written consent for material from their foals to be used for research. Seven of the 23 fetuses and foals were excluded because they were radiologically unremarkable, whereas 16 fetuses and foals were included because they had generalized or focal radiological changes.^28^ Included fetuses and foals were 0 to 150 days of age and comprised 9 colts and 7 fillies, with 11 Icelandic horses, 1 coldblooded trotting horse, 2 standardbred horses, and 2 warmblood horses (Supplemental Table S1).

The fetuses and foals were grouped by generalized and focal radiological changes (Supplemental Table S1) and assigned case numbers by ascending age within each group. Cases 1 and 2 had generalized changes in the CTB and TIII, whereas case 3 had generalized changes in both bones and focal changes in TIII. Cases 4 to 16 had focal changes in the CTB and/or TIII, consisting of uniformly hypodense defects in or near the ossification front, interpreted radiologically as osteochondrosis.^28^

### Sample Preparation

**Figure 1.**
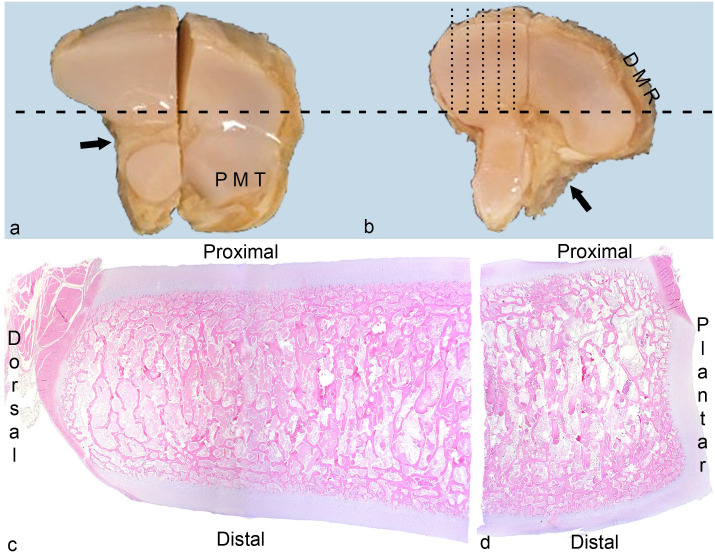
Methods. **(a)** Central tarsal bone, proximal view, horse. The tarsal bones were sawed into approximate medial and lateral halves, and each half was cut into a dorsal and a plantar quarter (dashed line). PMT, plantaro-medial tubercle. Black arrow, ligament fossa. Case 16. **(b)** Third tarsal bone, proximal view, horse. All bone quarters were cut into 2 to 3 mm-thick slabs in the parasagittal plane (dotted lines). DMR, dorso-medial ridge. Black arrow, ligament fossa. Case 16. **(c)** Third tarsal bone, dorso-medial quarter, horse. The proximal, dorsal, and distal aspects were evaluated separately. Case 4. Hematoxylin and eosin (HE). (**(d)** Third tarsal bone, plantaro-medial quarter, horse. The proximal, plantar, and distal aspects were evaluated separately. Case 4. HE.

Bones were initially selected for preparation based on identification of radiological changes in micro-CT scans (Supplemental Table S1). The CTB and TIII had been sawed into approximate medial and lateral halves for micro-CT (Fig. 1a).^28^ The bone halves were fixed in 4% phosphate-buffered formaldehyde for 48 hours and decalcified in 10% ethylenediaminetetraacetic acid. Each bone half was cut into a dorsal and a plantar quarter, and all quarters were cut into 2 to 3 mm-thick serial slabs in the parasagittal plane (Fig. 1b).

### Selection of Slabs for Histology

From cases 1 to 3 with generalized changes, 1 or 2 slabs located centrally within TIII were selected for histology. From cases 4 to 16 with radiological osteochondrosis, all slabs with focal irregularities at the cartilage-bone interface visible on the surface of the slab were selected for histology. Slabs without visible surface changes were also selected based on the location of defects in micro-CT scans, as follows: The micro-CT scan was divided into thirds and the macroscopic sample was divided into thirds. The third of the micro-CT scan that contained the lesion was identified and the slabs representing that third were selected for histology.

### Sectioning

After paraffin embedding, at least one 4- to 6-µm-thick section was prepared from the surface of each selected slab and stained with hematoxylin and eosin. The surface sections were inspected for changes that matched the radiological descriptions (Supplemental Table S1). If the surface sections were normal, additional sections were prepared 0.5 mm deeper into the slab up to 3 times, and 2 slabs were serial-sectioned (Supplemental Table S2). Once capture of a radiological lesion was confirmed within 1 slab, the remaining slabs from that case were not sectioned further. If there was matrix pallor in hematoxylin and eosin-stained sections, additional sections were prepared and stained with toluidine blue, and if there were perivascular neutrophils or suspicion of bacteria within cartilage canals, Gram-Twort sections were prepared.^33^

### Parameters Observed

Sections from all cases were systematically evaluated for the presence of tissues, cartilage canals, and pathological changes. Tissues and canals were evaluated in sections without focal lesions. Proximal, distal, dorsal, and plantar aspects were evaluated separately (Fig. 1c, d).

Recorded tissues included articular cartilage, growth cartilage, and fibrous (dense connective) tissue. Articular cartilage was defined as the superficial portion of the articular-epiphyseal cartilage complex adjacent to the synovial cavity. Growth cartilage was defined as cartilage organized in resting, proliferative, hypertrophic, and mineralized zones.^23^ Fibrous tissue included thin layers of irregular tissue covering cartilage or bone (ie, perichondrium or periosteum) and thicker bands or wider sheets of more regular ligaments at the proximal (Fig. 1) and distal ligament fossae of the CTB and TIII. No attempt was made at distinguishing between the listed fibrous tissues and joint capsule.

Cartilage canals in morphologically normal tissue were categorized as patent canals containing intact vascular luminae and viable endothelial and mesenchymal cells, and as chondrifying canals containing disintegrating vessels surrounded by chondrocyte-like cells.^7,23^ Patent canals that contained perivascular neutrophils were referred to as inflamed patent canals.^33^

Necrotic cartilage canals contained dilated or disrupted vascular luminae lined by pyknotic or karyolytic endothelial cells surrounded by necrotic perivascular cells.^7,23^ When necrotic canals contained considerable intensely eosinophilic-staining material, they were called eosinophilic streaks.^22^ Necrotic canals that contained perivascular neutrophils, with or without bacteria in Gram-stained sections were referred to as acute septic canals.^33^ Necrotic canals surrounded by coagulative-necrotic chondrocytes (ie, areas of ischemic chondronecrosis within growth cartilage) were diagnosed as osteochondrosis latens, whereas areas of ischemic chondronecrosis within the ossification front were diagnosed as osteochondrosis manifesta.^34^ Solid areas of ischemic chondronecrosis surrounded by bone on all margins were diagnosed as pseudocysts, and similar areas that contained dilated, fibrous tissue–lined cystic spaces were diagnosed as true cysts.^21^ This deviation from how true cysts are usually defined as having an epithelial lining was discussed in Olstad et al, 2015.^21^ Secondary reparative responses were recorded, including proliferation of adjacent viable canals and chondrocytes (synonyms: clusters, nests, chondrones, and clones) outside the proliferative zone of growth cartilage.^19,23,31^ Secondary responses in bone included accumulation of chondroclasts and formation of fibro-vascular granulation tissue.^19,23,31^ Necrosis of chondrocytes superficially within articular cartilage was interpreted as early osteoarthritis.^4,13^

The data analyzed in this study are available from the corresponding author.

## Results

Selected slabs (Supplemental Table S2) were translated from millimeters to percentage to indicate which regions were best represented across the ages, and these were from 50% to 80% of the axial-to-abaxial distance in the plantaro-medial quarter of the CTB, and from 40% to 60% of the same distance in the dorso-medial quarter of TIII (Supplemental Table S3).

### Tissues Present—Proximal and Distal Aspects

**Figure 2.**
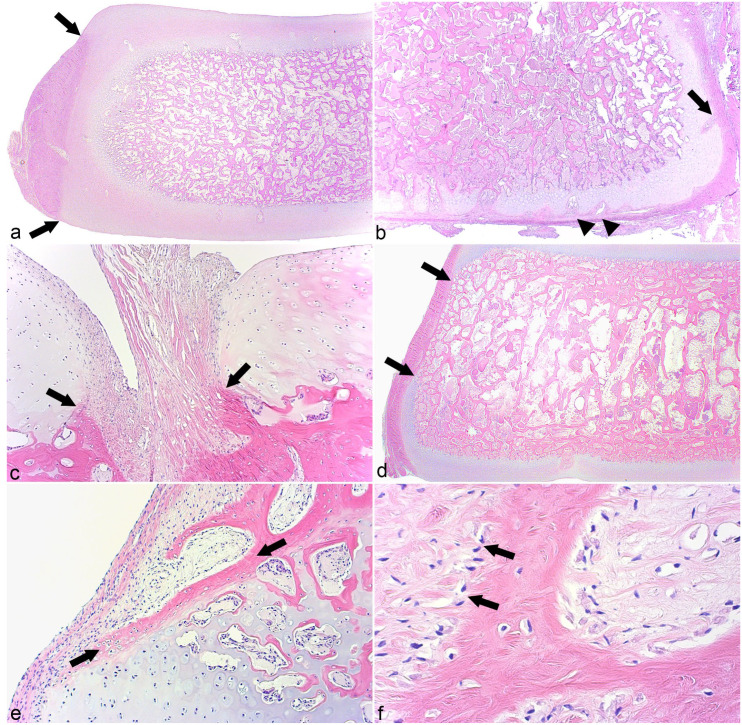
Tissues present. **(a)** Central tarsal bone, dorso-lateral quarter, horse. Toward the proximal and distal aspects, the ossification center is covered by growth cartilage and articular cartilage, whereas toward the dorsal aspect it is covered by growth cartilage and perichondrium (between arrows). Case 7. Hematoxylin and eosin (HE). **(b)** Third tarsal bone, plantaro-medial quarter, horse. The distal surface of the third tarsal bone is covered by a thin layer of fibrous tissue or “articular perichondrium.” Vessels enter growth cartilage canals from plantar perichondrium (arrow) and articular perichondrium (arrowheads). Case 7. HE. **(c)** Central tarsal bone, plantaro-medial quarter, horse. Ligament fibers (between arrows) are present on a small portion of the proximal aspect of the sample and merge directly with the ossification center. Case 16. HE. **(d)** Central tarsal bone, dorso-medial quarter, horse. The central portion of the dorsal aspect of the sample is covered by fibrous tissue (between arrows) that is in direct contact with bone, whereas growth cartilage remains toward the proximal and distal corners (above and below arrows). Case 4. HE. **(e)** Central tarsal bone, dorso-medial quarter, horse. The image shows a spicule of osteoid (between arrows) toward the dorso-distal corner of the bone. Below the spicule, there is endochondral ossification within growth cartilage. Above the spicule, there is intramembranous ossification within fibrous tissue. Case 12. HE. **(f)** Higher magnification of (**e)**. Toward the right side of the image, there are bone marrow spaces of primary spongiosa. Toward the left side of the image, there are osteoblasts (arrows) producing osteoid into fibrous tissue, that is, intramembranous ossification. Case 12. HE.

Tissues present are summarized in Supplemental Table S4. Ossification centers were absent or small in cases 2 and 3 described in the following, but in the remaining cases, the proximal and distal aspects of ossification centers tended to be covered by a deep layer of growth cartilage and a superficial layer of articular cartilage (Fig. 2a). In the youngest foals (0–34 days of age), growth cartilage zones were thick and chondrocytes were randomly oriented, whereas after 42 days of age, zones were thinner and chondrocytes were often oriented perpendicular to the underlying ossification front, sometimes in tall stacks resembling the physis (Supplemental Table S4). Two cases were slightly different: In parts of the distal aspect of TIII in case 1 and the entire plantar half of TIII in case 7, the growth cartilage was covered by a thin layer of fibrous tissue, referred to as “articular perichondrium” (Fig. 2b). In regions that corresponded to the ligament fossae (Fig. 1a, b), there was no cartilage, but rather ligament fibers merged directly with the ossification center (Fig. 2c). Proximally, ligament fibers tended to be present for small portions of the samples (Fig. 2c), whereas distally, they were sometimes present over much larger portions and sometimes the entire distal aspect of a slab, especially abaxially.

### Abaxial Aspect

In cases 1 to 3 and 5 to 7 that were up to 4 days of age, the dorsal and plantar (ie, abaxial aspect of each section) was covered by growth cartilage and perichondrium (Fig. 2a; Supplemental Table S4). In cases 4 and 8 to 13 that were up to 56 days of age, the abaxial aspect was covered partly by growth cartilage and perichondrium, and partly by a thick layer of fibrous tissue that was in direct contact with bone (Fig. 2d). In foals up to 56 days of age, the fibrous tissue was located at in the middle segment and growth cartilage remained toward the proximal and distal corners (Fig. 2d). With age, the proportion of fibrous tissue increased to 100% (Supplemental Table S4), such that in cases 14 to 16 that were between 105 and 150 days of age, the abaxial aspect was covered only by fibrous tissue. In some of the youngest foals, the connective tissue was somewhat heterogeneous and contained fibroblasts, remnant chondrocytes, and osteoblasts with osteoid; thus, bone formation occurred within the connective tissue itself. In older foals (7–150 days of age), the connective tissue contained only fibroblasts and in all of them, bone formed on the trabecular surfaces toward the fibrous tissue (ie, intramembranous ossification, Fig. 2e, f). These observations indicated that up to 4 days of age, growth occurred by endochondral ossification toward all aspects of the CTB and TIII. From days 4 to 56, abaxial growth occurred by both endochondral and intramembranous ossification, and from 105 days of age, abaxial growth occurred by intramembranous ossification only.

### Cartilage Canals

The growth cartilage of cases 1 to 10 that were up to 34 days of age consistently contained patent cartilage canals. Sections from some cases contained examples of chondrifying canals (Supplemental Table S4). The growth cartilage of cases 13 (56 days of age) and 15 (122 days of age) contained 2 patent canal stumps each, whereas canals were not seen in any section from cases 11 to 12 (42 and 47 days of age) or 14 and 16 (105 and 150 days of age, respectively).

**Figure 3.**
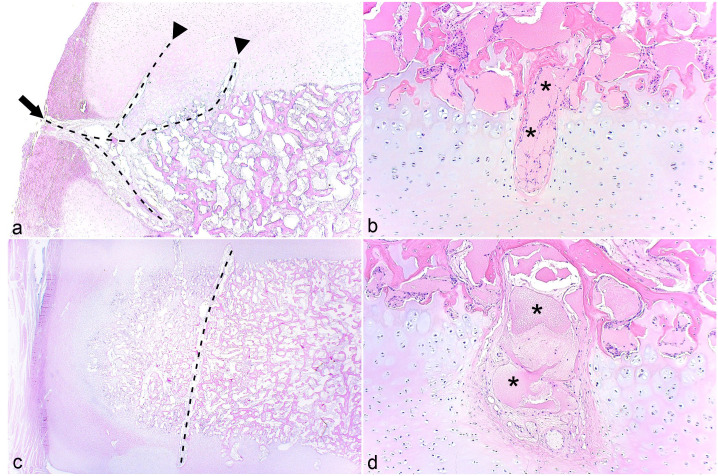
Cartilage canals. **(a)** Central tarsal bone, dorso-medial quarter, horse. A vessel (dashed line) enters growth cartilage canals from dorsal perichondrium (arrow) and courses toward the center of the bone. Two branches (arrowheads) turn to course vertically toward the proximal articular cartilage, and the midportion of the deepest branch courses into the subchondral bone at the peripheral corner of the sample. Case 6. Hematoxylin and eosin (HE). **(b)** Third tarsal bone, dorso-medial quarter, horse. Vessels (asterisks) midway between the periphery and center enter and exit a cartilage canal from subchondral bone and course perpendicular to the underlying ossification front. The canal is of the anticipated approximately 0.5 mm width. Case 4. HE. **(c)** Third tarsal bone, dorso-medial quarter, horse. A vessel (dashed line) can be followed from a canal toward the proximal aspect of the bone through the ossification center and into a canal toward the distal aspect, that is, a transverse vessel. Case 6. HE. **(d)** Central tarsal bone, dorso-medial quarter, horse. The image shows a patent canal that is considerably wider than the anticipated width, referred to as a “wide canal.” The canal contains the same structures as regular-width canals although some of the vascular luminae (asterisks) appear to be dilated. Case 4. HE.

From peripheral to central within samples, vessels were observed to enter growth cartilage canals from 4 sources: abaxial perichondrium (Figs. 2b, 3a), articular perichondrium (Fig. 2b), subchondral bone (Fig. 3b), and ligaments. When cut in longitudinal section, peripheral perichondrial vessels were observed to course toward the center of the bone, then turn to course vertically toward the articular cartilage on the proximal (Fig. 3a) or distal aspect. The midportion of vessels or branches coursed through subchondral bone at the peripheral corner of the sample (Fig. 3a). Vessels entering canals from subchondral bone midway between the periphery and center tended to course perpendicular to the underlying ossification front (Fig. 3b). Sometimes, a vessel could be followed from a canal toward one aspect of the bone through the ossification center and into a canal toward the opposite aspect of the bone (ie, constituted a transverse vessel, Fig. 3c). After entering cartilage, a low number of vessels made a 90° turn to form an L-shape, and occasional vessels made a complete 180° turn back into subchondral bone. Most patent canals were approximately 0.5 mm wide (Fig. 3b), but some canals midway between the periphery and center were considerably wider, measuring 1 to 2 mm in width and were referred to as “wide canals” (Fig. 3d, Supplemental Table S4). Wide canals contained the same structures as regular-width patent canals, although vascular luminae occasionally appeared dilated (Fig. 3d).

### Pathological Changes

All sections from 6 cases (4, 8 and 9, and 14–16) were normal, whereas histological changes were detected in sections from the remaining 10/16 (62.5%) cases (Supplemental Table S5).

### Cases 1 to 3 With Generalized Changes

The TIII of case 1 contained patches where the ossification front was smooth, matching the radiological description. Superficial to the smooth patches, there were foci with fewer cells, less hypertrophy, and more extracellular matrix than adjacent regions and the next cases up and down in age, indicating that the changes had occurred antemortem.

The TIII of case 2 contained a small mineralized focus and TIII of case 3 contained a small ossification center, both set within cartilage that was markedly thicker than in any other case, compatible with incomplete ossification. Chondrocytes and matrix outside of the lesioned area were normal in both cases. Central chondrocytes in the affected area were large, round, and only organized into growth cartilage zones immediately adjacent to the mineralized focus of case 2 or the ossification center of case 3. The central chondrocytes were nucleated, contained pale, sometimes foamy cytoplasm, and were surrounded by pale matrix. The focal defect in the ossification front of case 3 contained chondrocytes with the same morphology as the central chondrocytes. There were multiple canal profiles in the cartilage of cases 2 and 3, but their contents were consistently absent despite repeat and serial sectioning.

### Cases 5, 7, and 12 With Focal Changes Interpreted as Normal or Inconclusive

Case 5 was serial-sectioned until one of the 2 radiological defect lobes was captured. The lobe contained a patent vessel surrounded by viable chondrocytes and was therefore interpreted to represent a normal vascular dimple. In case 7, a small focus of pale matrix was captured, but the underlying ossification front was normal. The slab was serial-sectioned without revealing any further changes; thus, the evaluation was inconclusive. Sections from case 12 contained a large, circular nonossified area at the proximal margin of the dorso-medial ridge of TIII. The central part contained debris and the peripheral rim consisted of fibrous tissue. Adjacent canals and chondrocytes were normal, including in serial sections. The circular area could represent a true cyst or a contour that was irregular, but normal for the developing dorso-medial ridge. Due to a lack of age-matched comparisons, the evaluation was considered inconclusive.

### Cases 6, 10, 11, and 13 With Focal Changes Confirmed to Represent Disease

The radiological defect toward the distal aspect in the CTB of case 11 was not captured, but toward the same aspect, 2 adjacent slabs contained ~10 to 30 necrotic chondrocytes superficially within the articular cartilage that were interpreted as early osteoarthritis. In cases 6, 10, and 13, defects were captured that contained changes diagnosed as osteochondrosis and secondary repair responses, as listed in the approximate order of size, severity, and extent of secondary changes in Supplemental Table S6.

**Figure 4.**
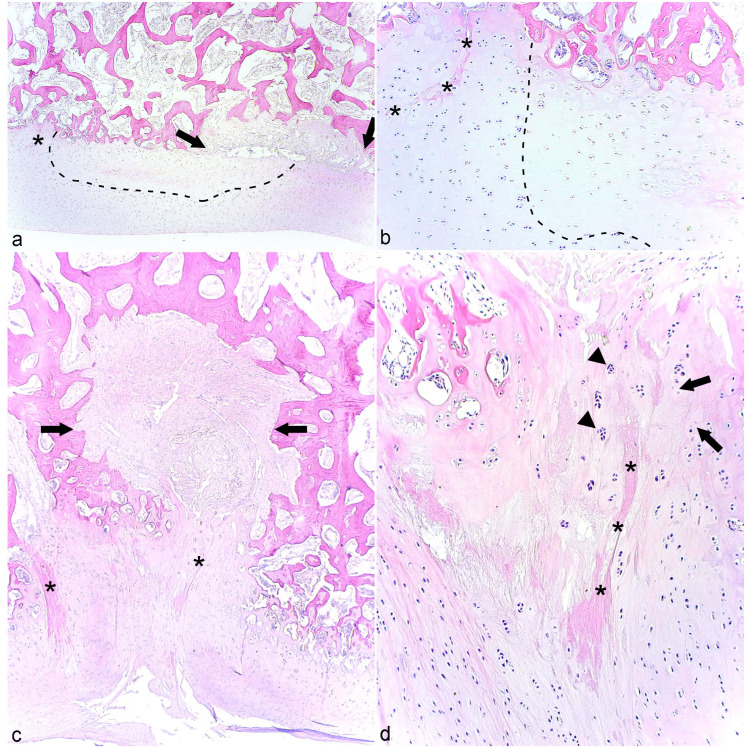
Osteochondrosis latens and pseudocysts. **(a)** Central tarsal bone, plantaro-lateral quarter, horse. There is a lesion consisting of a small bilobed area located partly within growth cartilage (within dashed line; greater magnification shown in (**b)**, same necrotic vessel labeled with asterisks in (**a)** and (**b)**, and partly within the ossification front (between arrows). The area in the ossification front consists of granulation tissue and is causing a small defect in subchondral bone, presumably representing the margin of the radiological cyst and, as it is mostly solid, it was characterized as a pseudocyst. Case 13. Hematoxylin and eosin (HE). **(b)** Higher magnification of (**a)**. The area in cartilage consists of necrotic chondrocytes (to right of dashed line) with a necrotic cartilage canal (asterisks), that is, osteochondrosis latens. Case 13. HE. **(c)** Central tarsal bone, dorso-medial quarter, horse. There is a lesion consisting of a central area of granulation tissue (between arrows) with eosinophilic streaks (asterisks) within the lesion and on the superficial margins toward cartilage. The granulation tissue contains dilated spaces, but is mostly solid and was therefore also characterized as a pseudocyst. Case 10. HE. **(d)** Higher magnification of (**c)**. An eosinophilic streak (asterisks) is associated with a low number of necrotic chondrocytes (arrows), that is, osteochondrosis latens and several clusters (arrowheads). Case 10. HE.

The micro-CT lesion in the CTB of case 13 consisted of a small, bilobed area located partly within growth cartilage and partly within the ossification front (Fig. 4a). The area in the cartilage was an osteochondrosis latens lesion (Fig. 4b). The lesion was associated with a shallow band of granulation tissue, causing a small defect in the subchondral bone (Fig. 4a), presumably representing the margin of the radiological cyst. As the lesion was mostly solid, it was characterized as a pseudocyst (Supplemental Table S3). The captured lesion corresponding to the radiological cyst in TIII of case 10 was larger and contained a central area of granulation tissue (Fig. 4c), with eosinophilic streaks and occasional necrotic chondrocytes centrally within the lesion and superficially within the lesion toward the overlying cartilage, that is, osteochondrosis latens (Fig. 4d). The eosinophilic streaks were associated with several chondrocyte clusters (Fig. 4d). Although it contained some dilated spaces, the majority of the granulation tissue was solid (Fig. 4c) and the lesion was therefore also characterized as a pseudocyst (Supplemental Table S6).

**Figure 5.**
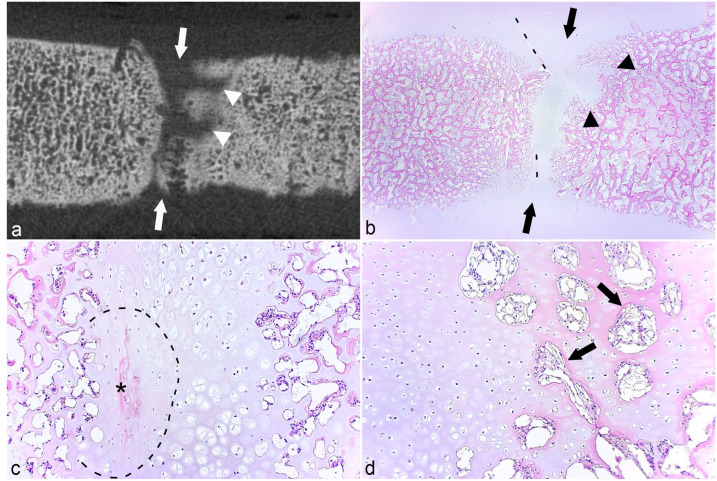
Cylindrical osteochondrosis manifesta and reparative endochondral ossification. **(a)** Third tarsal bone, middle cylindrical defect, horse. There is a cylindrical defect (between arrows) with smaller defects extending sideways (arrowheads), radiologically interpreted to correspond to failure of a transverse vessel. Case 6. Micro-computed tomographic image reprinted with permission from Sigurdsson et al.^28^ **(b)** Central tarsal bone, dorso-lateral quarter, horse. There is a linear area of retained growth cartilage in bone (between arrows) centered on portions of necrotic cartilage canals (dashed lines) that are oriented in the proximo-distal direction, interpreted as failure of transverse vessels. A smaller area of cartilage (between arrowheads) extends sideways, compatible with a failed side branch. Case 6. Hematoxylin and eosin (HE). **(c)** Central tarsal bone, dorso-lateral quarter, horse. There is an eosinophilic streak (asterisk) surrounded by a narrow zone of ischemic chondronecrosis (within dashed line; osteochondrosis manifesta) similar to articular osteochondrosis. The rest of the retained cartilage consists of viable, hypertrophic chondrocytes similar to physeal osteochondrosis. Case 6. HE. **(d)** Central tarsal bone, dorso-lateral quarter, horse. A canal has proliferated in the cartilage adjacent to the lesion in **(b)**, and luminae have intensely eosinophilic-staining margins (arrows) suggestive of osteoid, indicating that they are in the process of forming a separate center of reparative ossification. Case 6. HE.

The CTB and TIII (Fig. 5a) of case 6 contained 2 and 3 cylindrical radiological defects, respectively. The lesions were captured in sections from the CTB where they corresponded to linear areas of retained growth cartilage centered on necrotic canals (Fig. 5b). When sectioned tangentially, portions of the necrotic canals were oriented in the proximo-distal direction and were interpreted to represent failure of transverse vessels. There were also smaller areas of retained cartilage extending sideways, which were compatible with failed side branches (Fig. 5b). The necrotic canals were either eosinophilic streaks (Fig. 5c) or contained debris that was suspicious of a septic process, but did not have identifiable neutrophils or bacteria at the time of sampling. Both the CTB and TIII contained inflamed patent canals. An osteochondrosis latens lesion was captured in TIII, possibly representing sectioning through the superficial, peripheral margin of one of the larger, noncaptured cylindrical defects. The cartilage within the captured cylindrical defect in the CTB included both osteochondrosis manifesta and retained, viable hypertrophic chondrocytes (Fig. 5c) and therefore had similarities with both articular (ischemic-necrotic) and physeal (retained-hypertrophic) osteochondrosis. Viable canals adjacent to lesions had proliferated and had intensely eosinophilic margins suggestive of osteoid (Fig. 5d), indicating that they were in the process of forming separate centers of reparative endochondral ossification.

**Figure 6.**
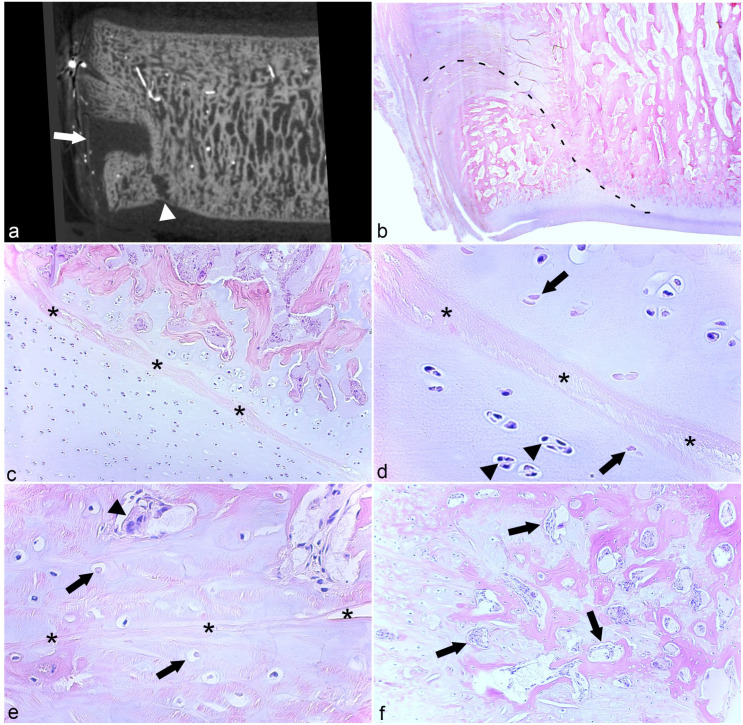
Shelf and groove osteochondrosis manifesta and reparative intramembranous ossification. **(a)** Third tarsal bone, dorso-lateral quarter, horse. There is a large, dorsal shelf defect (arrow) that is connected by a narrow stalk to a distal groove defect (arrowhead). Case 13. Micro-computed tomographic image reprinted with permission from Sigurdsson et al.^28^ **(b)** Third tarsal bone, dorso-lateral quarter, horse. In sections, it was possible to follow horizontally oriented remnants of a necrotic canal from the periosteum into the dorsal shelf in the same section as remnants coursing vertically into the distal groove that were interpreted to represent failure of a vertically turning vessel (dashed line). Portions of this necrotic canal are shown at higher magnification in (**c)** to (**e)**. Case 13. Hematoxylin and eosin (HE). **(c)** Third tarsal bone, dorso-lateral quarter, horse. In the distal groove, there is an eosinophilic-streak necrotic canal (asterisks). **(d)** Higher magnification of (**c)**. The eosinophilic streak is surrounded by a low number of necrotic chondrocytes (arrows), that is, osteochondrosis manifesta, with adjacent chondrocyte clusters (arrowheads). Case 13. HE. **(e)** Third tarsal bone, dorso-lateral quarter, horse. The dorsal shelf primarily contains granulation tissue and within it there are remnants of necrotic cartilage canals (asterisks) and necrotic chondrocytes (arrows), that is, osteochondrosis manifesta. There is also a chondroclast (arrowhead), presumably engaged in the removal of necrotic cartilage. Case 13, HE. **(f)** Adjacent to (**e)**. Within the granulation tissue, there are osteoblasts (arrows) with osteoid, indicating repair by intramembranous ossification. Case 13. HE.

In micro-CT scans, the large shelf and groove defects dorso-laterally in TIII of case 13 were connected by a narrow stalk (Fig. 6a) and, in histological sections, it was possible to follow remnants of a necrotic vessel from the periosteum, through the dorsal shelf, and vertically into the distal groove (Fig. 6b–e). The shelf and groove were present in multiple adjacent slabs inferring that the necrotic vessel also had circumferential branches, but such branches were not easy to follow between slabs cut in the parasagittal plane. The distal groove defect and cartilage superficial to it contained an osteochondrosis manifesta lesion with adjacent chondrocyte clusters (Fig. 6c, d). The dorsal shelf contained mainly granulation tissue, but within it there were both areas of osteochondrosis manifesta (Fig. 6e) and reparative intramembranous ossification (Fig. 6f).

## Discussion

The main achievement of this study was a description of tissues, cartilage canals, and lesions present during development of the CTB and TIII.

### Tissues Present

The description of tissues proved useful because some anticipated tissues were absent and some unexpected tissues were present. Based on previous reports,^14,31^ we were expecting all aspects of the CTB and TIII to be covered by growth cartilage, but from 105 days of age, the abaxial aspect was covered only by fibrous tissue. Ligament fibers were anticipated,^28^ but it was surprising that they could cover the entire distal aspect of plantar slabs. Articular perichondrium was entirely unexpected, and may represent some form of fetal remnant. The most likely reason why these observations have not been made before is that sampling was different between studies. In studies of incomplete ossification,^14,15^ foals tend to be sampled at a young age when abaxial growth cartilage is still present, whereas in studies of distal tarsal osteoarthritis,^3,4,11,31^ sampled foals have usually been older than 3 months of age. Depending on breed, the earliest signs of osteoarthritis are often described to appear dorso-medially or dorso-laterally,^4^ meaning the plantar aspect and ligament fossae are rarely sampled. One of the most important realizations during this study and the previous micro-CT study^28^ is that early osteochondrosis defects can frequently be found toward the plantar aspect of the CTB and TIII. Plantar wedging has also been associated with more severe osteoarthritis than dorsal wedging.^29^ It may therefore become more important for pathologists to sample and know the plantar anatomy of the CTB and TIII in future. In this context, it should already be noted that there was individual variation in the number of ligament fossae present that were not explained by age or breed.^28^ We recommend taking photographs of the intact bones before sectioning, so that it is always possible to go back and see which ligament fossae to expect in a particular individual.

Another major implication of this study is that, from 105 days of age, abaxial growth must occur by intramembranous rather than endochondral ossification. The largest current sampled bones (case 15, standardbred horse) were informally compared with mature teaching specimens (1 thoroughbred horse and 1 warmblood horse with closed growth plates) and the results indicated that the young bones were due to grow at least 0.8 cm in the dorso-plantar direction and approximately 1.5 cm in the latero-medial direction. After the switch to intramembranous ossification, it will no longer be possible to develop any delay in endochondral ossification/osteochondrosis. It is possible to develop disturbance of intramembranous ossification as exemplified in mandibular sutures (eg, prognathism),^2,15^ but whether a similar disturbance is responsible for any of the developmental diseases seen in tarsal bones is currently unknown.

### Cartilage Canals

The number of patent cartilage canals in the CTB and TIII decreased markedly between 34 and 56 days of age, but this study was subject to some limitations. Not all regions were sampled from all foals, and foals represented a hospital population where they tend to cluster around certain ages. The 2 vessel stumps in case 15 at 122 days of age suggest that vessels are present for longer in the CTB than the tibia and talus where vessels reportedly regress at less than or equal to 70 days of age.^6,24^ However, slightly different perfusion and vessel visualization techniques were used in previous studies;^6,24^ thus, the difference may not have been quite as long as 52 days if the tibia and talus technique for studying blood supply had been used for the CTB. It would be useful to add data from more 34 to 150 days of age foals to delineate the process more precisely in future, but for now, the results indicate that vascular regression from the growth cartilage of the CTB and TIII is complete sometime between 122 and 150 days of age.

In terms of patent canals, a new morphology of wide canals was observed, the main significance of which may be that it must be distinguished from vessels that have failed and become dilated, that is, early osteochondrosis.^21^ The other main observation about patent vessels that entered cartilage canals of the CTB and TIII was that they traversed the 2 tissue junctions that have previously been implicated in vascular failure: the junction between growth cartilage and bone^24,34^ and between growth cartilage and perichondrium at ligament sites.^10^ Failure of the blood supply to growth cartilage may therefore occur by similar mechanisms in distal tarsal bones as in the talus^24^ and long bones.^24,34^

### The Challenge of Capturing and Diagnosing Changes

The rate of capturing any changes in histological sections was 62.5% (10/16 cases), which seems low, considering that when a defect was identified during previous tibiotalar micro-CT, it was always captured in sections.^17^ In the tibiotalar study,^17^ the sides of the sample blocks represented saw cuts that were identical to the sides of the scans and, in hindsight, more soft tissue should have been removed before selection of the current slabs. Selection is clearly critical when considering that, even when sectioning the current 2 to 3 mm-thick slabs at up to 4 levels of depth, there would be greater than or equal to 80 sections per level and, at greater than or equal to 50 µm,^28^ the smallest radiological defects would be present in just ~13 of the sections. The only way to guarantee the capture of every defect would be to section at less than 50 µm intervals through entire slabs, something that was financially impossible. Capturing small defects may be clinically irrelevant because small lesions are likely to resolve without becoming significant.^20^ However, the challenge remains relevant in research, especially when studying a disease process that can begin with only 1 canal and a few surrounding cells.^20^ If we were to repeat this study, we would try to use stereotactic localization^13^ and defect-oriented planes of section to increase the rate of capture.

The radiological defect captured in case 5 was diagnosed as a normal vascular dimple because it contained viable vessels and chondrocytes.^17^ This seems to suggest that a false-positive diagnosis of a normal feature as a lesion occurred during the micro-CT evaluation.^28^ However, only 1 lobe was captured in sections, whereas the radiological defect was bilobed. The possibility therefore remains that it was the histological diagnosis that was a false-negative due to incomplete representation of the lesion. If it had been located in the middle of the proximal or distal aspect, the defect was unlikely to represent a normal dimple because dimples toward these aspects were small, single-lobe, and occurred as several together in a region,^28^ but the defect in case 5 was located at the periphery and it is not known whether peripheral dimples can be more irregular and still be normal. Both cases 5 and 7 were serial-sectioned and the pale focus in case 7 strongly resembled tangential sectioning through the margin of a lesion,^6,24^ but further sectioning revealed more normal tissue, thus it appeared to move away from any additional lesions. The circular nonossified area in case 12 was so large that it is possible that peripheral margins were incompletely represented in the sections, but chondrocytes at the deep margins were normal. However, because it was so large, if the area did represent a cyst,^21^ it would have been chronic, meaning early pathological changes could have resolved by the time of sampling. Similar to case 5, if it had been in any other location, the lesion in case 12 would have been a cyst, but it was located at the dorso-medial ridge of TIII during the age window when ridge formation is most active.^28^ The tissue in the opposite direction from the serial sections in case 7 was not available for further sectioning, but cases 5 and 12 represent diagnostic challenges that may be resolved by examining material from appropriate age-matched comparisons in future to determine how irregular they can be and still be normal.

### Generalized Changes in Cases 1 to 3

The dam of case 1 died unexpectedly at term, and case 1 had cellular changes compatible with antemortem disease, not artifact. There are plausible explanations for how the dam could have suffered subclinical micronutrient imbalance^2,14,15^ or disease for long enough to cause skeletal changes in the fetus before culminating in the death of both. However, this is the first time in decades of research that the authors have encountered such changes, thus they are unlikely to play any major role in routine diagnostics.

Cases 2 and 3 had absent or undersized ossification centers within cartilage that was markedly thicker than in any other case; a morphology widely recognized among equine clinicians as incomplete ossification.^1,9,14^ The range for normal equine gestation is very wide,^1,2^ and the term “incomplete ossification” is applied to premature, dysmature, and normal foals.^2,9,14^ Case 2 was reportedly 6 weeks premature, and the undersized ossification centers may be normal for that stage of gestation in which case there is no reason to look for causal lesions.^1^ Conversely, case 3 was not premature and had contracted tendons and incomplete carpal ossification, meaning there is every indication to look for preventable causal lesions, for example, hypothyroidism.^2,14,15^ The main histological feature of cases 2 and 3 was loss of the contents of cartilage canals that may represent sectioning artifact (case 2), but we also have to consider that it could represent primary lesions (case 3). Poulos^26^ and Pool^25^ suggested that weight-bearing on immature cartilage caused a “decrease in blood supply,” leading to incomplete ossification. If weight-bearing was the true cause, the condition would be expected to occur symmetrically, but this is not always the case.^9^ In long bones, cartilage canal vessels are present before secondary ossification centers and initiate their formation.^5,23^ The empty canal profiles in case 3 potentially indicate that the arterial source to central vessels failed before they were due to initiate formation of the ossification centers in the CTB and TIII, causing canal contents to shrink and dropout. Vascular failure^18^ is a better causal explanation for incomplete ossification than weight-bearing^25,26^ because the condition would occur variably in the CTB and/or TIII^29^ of 1 or both hind limbs,^9^ depending on exactly which nutrient arteries failed. Once vessels have been compromised, Poulos,^26^ Pool,^25^ and the documented pathogenesis of articular osteochondrosis^18,19^ agree on how the condition eventually progresses: necrotic canals become recanalized, the growth cartilage is revascularized, and ossification resumes. The focal defects in case 3 are most compatible with resumed central ossification advancing around more peripheral chondrocytes that remain devascularized for longer because they are supplied by a different, more peripheral and not yet recanalized arterial source.

### Focal Changes in Cases 6, 10, 11, and 13

In cases 6, 10, and 13, defects were captured that contained cartilage centered on necrotic vessels, confirming they represented vascular failure.^6,19,24^ The cysts in cases 10 and 13 were compatible with failure of perpendicular (vertical) vessels entering growth cartilage from subchondral bone midway between the periphery and center (Fig. 3b),^17,28^ the cylindrical defects in case 6 were compatible with failure of transverse vessels (Fig. 5),^28^ and the shelf and groove defects in case 13 represented failure of vessels entering canals at the dorsal perichondrium, and then turning to course vertically toward the distal articular cartilage (Fig. 6).^24,28^

In cases 6, 10, and 13, necrotic canals included eosinophilic streaks more commonly described in conjunction with physeal^22^ than articular osteochondrosis.^6,24^ Cartilage surrounding necrotic vessels in case 6 included areas of ischemic-necrotic cartilage, typical of articular osteochondrosis,^6,24^ and retained-hypertrophic cartilage, typical of physeal osteochondrosis.^22^ Watrous et al^31^ also described distal tarsal osteochondrosis lesions as both “augmentation of the hypertrophied layer” and chondrocyte necrosis. Together, these observations support that distal tarsal osteochondrosis has similarities with both articular^6,24^ and physeal^22^ osteochondrosis of long bones. Following vascular failure, it is only chondrocytes outside of the diffusion distance from alternative sources that undergo ischemic necrosis.^7,20^ The difference between physeal and articular osteochondrosis has been explained as follows:^22^ Epiphyseal vessels tend to be organized as diverging end arteries,^24^ where the distance to intact neighbors increases as they diverge and eventually exceeds maximal diffusion distance, resulting in necrosis. Physeal vessels tend to be organized as parallel end arteries^32^ and diffusion distance to intact neighbors remains constant, meaning chondrocytes are able to adapt and survive, but arrest in hypertrophy, presumably due to missing some circulating cue to complete the ossification process.^22^ Watrous et al^31^ and the current lesions confirm that both things can happen simultaneously in different parts of the same lesion. Whether vascular failure results in necrosis or retention is likely to be governed by the diffusion distance to alternative sources in all 3 categories of physeal, epiphyseal, and cuboidal bone growth cartilage.

Ischemic necrosis stimulates proliferation of intact vessels and formation of centers of reparative ossification within growth cartilage,^17,19,24^ as seen adjacent to the lesion in case 6. Case 6 was only 3 days of age and ossification may advance past the reparative centers, causing them to disappear.^17^ However, reparative centers that arise late in growth when there is little remaining ossification potential could persist as foci that protrude above the rest of the cartilage-bone interface. This is relevant because such protrusions potentially overlap with changes that have been proposed to represent early osteoarthritis because they were identified in young, heritably predisposed horses.^12,13^

Once osteochondrosis lesions become surrounded by the ossification front, they initiate formation of granulation tissue as seen in cases 10 and 13, and the granulation tissue is capable of repair through intramembranous ossification,^17^ as seen in case 13. Loading of incompletely ossified bones is often considered to cause collapse, wedging,^29^ and angular limb deformity.^16^ The lesion in case 13 leads us to suggest a previous alternative suggestion from the carpus: that wedging may represent a consequence of osteochondrosis.^14^ At 56 days of age, the dorsal part of TIII in case 13 should consist of growth cartilage undergoing endochondral ossification. Instead, a large portion of it was replaced with granulation tissue undergoing intramembranous ossification. If intramembranous ossification of granulation tissue is slower than endochondral ossification, the dorsal part will grow less than the plantar part in the same time and TIII will become wedged.

In case 11, chondrocyte necrosis was detected superficially in the articular cartilage. Articular cartilage survives by diffusion and does not have any blood supply, thus the necrosis did not represent vascular failure. However, the necrosis was located toward the same distal aspect as where the osteochondrosis defect that the slabs were selected to capture was located, and a relationship between the necrosis and uncaptured defect may have existed. Previously, it was suggested that load dissipation might be altered superficial to an ossification front that is uneven due to indented osteochondrosis defects and protruding repair responses, and that this might produce local strain peaks leading to articular chondrocyte necrosis.^28^ Articular chondrocyte necrosis is another change that has been proposed to represent early distal tarsal osteoarthritis.^4,12,13^

In future, it would be ideal to detect vascular failure, chondrocyte necrosis, and retention and repair responses simultaneously by a modality that can be used longitudinally in vivo (eg, high-field MRI)^12^ as this could enable monitoring of the proposed pathogenesis and sequelae of distal tarsal osteochondrosis, including osteoarthritis.

In conclusion, the CTB and TIII grew by both endochondral and intramembranous ossification. The blood supply to the growth cartilage of the CTB and TIII regressed between 122 and 150 days. Radiological osteochondrosis defects represented vascular failure with chondrocyte necrosis and retention, or a combination of articular and physeal osteochondrosis.

## Supplemental Material

sj-pdf-1-vet-10.1177_03009858231185108 – Supplemental material for Osteochondrosis in the central and third tarsal bones of young horses Click here for additional data file. Supplemental material, sj-pdf-1-vet-10.1177_03009858231185108 for Osteochondrosis in the central and third tarsal bones of young horses by Kristin Olstad, Stina Ekman, Sigriður Björnsdóttir, Cathrine T. Fjordbakk, Kerstin Hansson, Sigurdur F. Sigurdsson and Charles J. Ley in Veterinary Pathology
