# Supplementary material for: Osteochondrosis in the central and third tarsal bones of young horses
Source: Vet Pathol. 2023 Jul 11;61(1):74–87. doi: 10.1177/03009858231185108 (PMC10687793; doi:10.1177/03009858231185108)
Supplement: sj-pdf-1-vet-10.1177_03009858231185108 – Supplemental material for Osteochondrosis in the central and third tarsal bones of young horses [file sj-pdf-1-vet-10.1177_03009858231185108.pdf]

Supplemental Materials

Osteochondrosis in the central and third tarsal bones of young horses

Kristin Olstad, Stina Ekman, Sigriður Björnsdóttir, Cathrine T. Fjordbakk, Kerstin Hansson, Sigurdur F. Sigurdsson, Charles J. Ley

Supplemental Table S1. Cases and radiological changes

Case	Generalized or focal changes	Age	Micro-CT case and lesion[a]	Bone	Location	Radiological changes	Sex	Breed	Cause of death or euthanasia	Systemic conditions; medications
1	Generalized	0 days	1	CTB and TIII	Generalized	Coarse, patchy defects in ossification front and bone marrow	Male	Icelandic horse	Mare died, full-term fetus	N/a
2	Generalized	1 day	3	CTB	Generalized	No ossification center	Male	Icelandic horse	Six weeks premature	N/a
				TIII	Generalized	Small mineralized focus				
3	Generalized and focal	1 day	15	CTB	Generalized	Small mineralized focus	Male	Standardbred horse	Congenital contracted forelimb tendons and incomplete carpal bone ossification	Oxytetra-cycline, penicillin, gentamicin
			15q	TIII	Generalized and dorsal, lateral and plantar focal defects	Small ossification center with three large focal defects				
4	Focal	0 days	2a	TIII	Peripheral, plantar, medial	Small focal defect	Male	Icelandic horse	Stillborn at term, large	N/a
			2u	TIII	Peripheral, dorso-medial	Large ovoid defect				
			2v	CTB	Peripheral, dorso-medial	Large protruding ossification repair tube				
5	Focal	1 day	4g	CTB	Peripheral, plantar, medial	Small bilobed defect	Male	Icelandic horse	Acute diarrhea, hemorrhagic	*E. coli* cultured
6	Focal	3 days	16s (partly kissing with lesion 16t)	CTB	Midway,[b] both proximal and distal, both medial and lateral	Two large cylindrical defects	Female	Warmblood riding horse	Ten days premature	*E. coli* cultured

			16t	TIII	Midway, both proximal and distal, both medial and lateral	Three large cylindrical defects				
7	Focal	4 days	6r	CTB	Peripheral, plantar, lateral	Small bilobed defect with repair ossification centers	Female	Icelandic horse	Premature	N/a
8	Focal	7 days	7c	TIII	Peripheral, plantar, lateral	Very small ovoid defect	Female	Icelandic horse	Separated from dam, starved	N/a
9	Focal	31 days	8h	CTB	Peripheral, plantar, medial	Two medium-sized defects near each other	Male	Icelandic horse	Diarrhea, hemorrhagic	N/a
10	Focal	34 days	20i (kissing lesion with 20j)	CTB	Midway, distal, medial	Medium cyst, kissing with 20j	Female	Coldblooded trotting horse	Colic; presumed impaction, secondary entero-colitis	*E. coli* and *Enterococcus* sp. Cultured; flunixin meglumine.
			20j	TIII	Midway, proximal, medial	Two medium to small cysts				
11	Focal	42 days	9e	CTB	Peripheral, distal, medial	Small multi-lobulated defect	Female	Icelandic horse	Cervical spine fracture	N/a
12	Focal	47 days	10b	CTB	Peripheral, plantar, lateral	Small ovoid defect	Male	Icelandic horse	Inexperienced dam, foal starved	N/a
			10d	CTB	Peripheral, dorso-medial	Small ovoid defect				
			10n	TIII	Peripheral, abaxial, medial	Small multi-lobulated defect				
13	Focal	56 days	22k (kissing lesion with 22l)	CTB	Midway, distal, medial	Medium cyst, kissing with 22l	Female	Warmblood riding horse	Perforated gastric ulcer, secondary entero-colitis	Endotoxemia; meloxicam, sucralfate.
			22l	TIII	Midway, distal, medial	Large cyst				
			22m	CTB	Peripheral, plantar, medial	Small multi-lobulated defect				
			22w (connecting stalk with 22x)	TIII	Peripheral, dorso-lateral	Large dorso-lateral shelf defect				
			22x	TIII	Midway, dorso-lateral	Large dorso-lateral groove in distal aspect, connected with 22 w				
14	Focal	105 days	11p	CTB	Peripheral, plantar, medial	Medium multi-lobulated defect	Male	Icelandic horse	Congenital thoracic scoliosis	N/a
15	Focal	122 days	23f	CTB	Peripheral, plantar, medial	Small triangular defect	Male	Standardbred horse	Nasal reflux of milk from birth;	Bacteria visible in lung

									esophageal dilatation and secondary broncho-pneumonia	sections, not cultured
16	Focal	150 days	12o	CTB	Peripheral, plantar, medial	Medium multi-lobulated defect	Female	Icelandic horse	Unknown	N/a

[a]Numbers and letters refer to the lesion identification numbers in Sigurdsson SF, Olstad K, Ley CJ, Bjornsdottir S, Griffiths DJ, Fjordbakk CT. Radiological, vascular osteochondrosis occurs in the distal tarsus, and may cause osteoarthritis. Equine Vet J. 2022;54: 82-96. [b]Midway: midway between periphery and center. Abbreviations: CT, computed tomography. CTB, central tarsal bone. N/a, not applicable. TIII, third tarsal bone.

Supplemental Table S2. Selected bones, slabs, sections and stains

	Central tarsal bone, 12/16 selected				Third tarsal bone, 10/16 selected			
Case	Available slabs[a]	Slabs sectioned	Sectioning rounds and depths	Number of sections	Available slabs	Slabs sectioned	Sectioning rounds and depths	Number of sections
1	19	N/a	N/a	N/a	21	2	2 rounds, 2 depths	5 HE, 1 TB
2	16	N/a	N/a	N/a	16	1	2 rounds, 1 depth	4 HE, 1 TB
3	25	N/a	N/a	N/a	20	2	2 rounds, 2 depths	4 HE, 1 TB
4	18	3	3 rounds, 3 depths	11 HE, 1 TB	20	4	3 rounds, 4 depths	16 HE
5[b]	22	3	3 rounds, 2 depths	12 HE, 1 GT	21	N/a	N/a	N/a
6	24	4	2 rounds, 2 depths	11 HE, 3 GT	22	2	2 rounds, 2 depths	5 HE
7[c]	16	4	3 rounds, 1 depth	11 HE, 1 TB	16	2	2 rounds, 1 depth	4 HE
8	18	N/a	N/a	N/a	17	2	3 rounds, 4 depths	10 HE
9	21	3	3 rounds, 1 depth	11 HE	22	N/a	N/a	N/a
10	22	3	3 rounds, 3 depths	10 HE	23	4	2 rounds, 1 depth	5 HE, 1 GT
11	21	2	3 rounds, 2 depths	10 HE, 1 TB	20	N/a	N/a	N/a
12	21	4	3 rounds, 2 depths	14 HE	22	2	2 rounds, 1 depth	4 HE
13	22	5	3 rounds, 3 depths	16 HE	22	4	3 rounds, 3 depths	14 HE
14	20	2	3 rounds, 2 depths	8 HE	18	N/a	N/a	N/a
15	22	3	3 rounds, 2 depths	11 HE	24	N/a	N/a	N/a
16	20	3	3 rounds, 2 depths	11 HE, 1 TB	18	N/a	N/a	N/a
Sum	327	43 slabs		144 sections	322	28 slabs		75 sections

[a]Bones were wider dorsally, thus there were more slabs from dorsal than plantar bone quarters, resulting in odd numbers. [b]An additional 11 HE-stained serial sections were available from one slab in foal 5. [c]An additional 12 HE-stained serial sections were available from one slab in foal 7. Abbreviations: GT, Gram TWORT. HE, hematoxylin and eosin. N/a, not applicable. TB, toluidine blue.

Supplemental Table S3. Regions represented in study, translated from axial-to-abaxial millimeters to percentage to correct for variable size

			Number of times 10% increment represented in sections[a]									
Bone	Quarter	Number sectioned	0-10	10-20	20-30	30-40	40-50	50-60	60-70	70-80	80-90	90-100
CTB, 12/16 selected	Plantaro-medial	8	0	0	2	3	5	6	6	6	1	1
	Dorso-medial	5	1	1	0	1	2	4	4	2	2	2
	Plantaro-lateral	3	1	1	1	2	2	1	1	2	3	3
	Dorso-lateral	2	1	1	1	2	2	1	1	1	1	1
TIII, 10/16 selected	Dorso-medial	8	1	1	1	2	4	4	3	2	1	1
	Plantaro-medial	4	0	0	1	1	1	2	2	1	0	0
	Dorso-lateral	3	0	0	0	0	1	1	1	2	0	0
	Plantaro-lateral	1	1	1	1	1	1	1	1	0	0	0

[a]0%: midline. 100% most abaxial margin.

CTB, central tarsal bone; TIII, third tarsal bone

Supplemental Table S4. Tissues and cartilage canals present

**Case**	**1**	**2**	**3**	**4**	**5**	**6**	**7**	**8**	**9**	**10**	**11**	**12**	**13**	**14**	**15**	**16**
**Age in days**	**0**	**1**	**1**	**0**	**1**	**3**	**4**	**7**	**31**	**34**	**42**	**47**	**56**	**105**	**122**	**150**
**Bone**	**TIII**	**TIII**	**TIII**	**CTB TIII**	**CTB**	**CTB TIII**	**CTB TIII**	**TIII**	**CTB**	**CTB TIII**	**CTB**	**CTB TIII**	**CTB TIII**	**CTB**	**CTB**	**CTB**
Growth cartilage on all aspects	+	+	+	-	+	+	+	-	-	-	-	-	-	-	-	-
Abaxial aspect[a] partly fibrous tissue	-	-	-	+	-	-	-	+	+	+	+	+	+	+	+	+
Percentage growth cartilage: fibrous tissue	N/a	N/a	N/a	60:40 60:40	N/a	N/a	N/a	10:90	20:80	0:100 50:50	50:50	10:90 0:100	10:90 0:100	0:100	0:100	0:100
Ossification on abaxial aspect	EC	N/a	EC	EC IM	EC	EC	EC	EC IM	EC IM	EC IM	EC IM	EC IM	EC IM	IM	IM	IM
Growth cartilage zones	All + but HTZ and PFZ patchy	No ossification center	All + small center only	All + some perpendicular	All + some perpendicular	All + random oriented	All + mostly oblique oriented	All + mostly oblique oriented	All + mostly perpendicular	All + oblique some stacks	All + mostly perpendicular	All + quite thin	All + clearly perpendicular	All + PFZ thin, mostly perpendicular	All + PFZ thin, mostly perpendicular	All + PFZ discontinuous mostly perpendicular
Average number of canals on proximal/abaxial/ distal aspect	5/6/5	Too many to count	Too many to count	0/2/1, 1/3/2	2/6/3	8/7/4, 7/5/8	9/5/1, 5/3/9	2/2/3	1/4/1	1/0/1	0/0/0	0/0/0	0/2/0, 0/0/0	0/0/0	0/0/2	0/0/0
Patent canals, regular width	+	+	+	+	+	+	+	+	+	+/-	-	-	+	-	+	-
Patent canals, extra wide	+	-	-	+	+	+	+	+	-	-	-	-	-	-	-	-
Chondrifying canals	-	-	-	-	-	+	+	-	+	+	-	-	-	-	-	-

[a]Abaxial aspect: dorsal or plantar aspect. Abbreviations: CTB, central tarsal bone. EC, endochondral ossification. HTZ, hypertrophic zone. IM, intramembranous ossification. N/a, not applicable. PFZ, proliferative zone. TIII, third tarsal bone.

Supplemental Table S5. Histological changes detected and radiological defects captured

Case	Generalized or focal changes	Micro-CT case and lesion[a]	Bone	Radiological changes	Captured in sections	Interpretation
1	Generalized	1	TIII	Coarse, patchy defects in ossification front	Yes	Intra-uterine disease
2	Generalized	3	TIII	Small mineralized focus	Yes	Incomplete ossification
3	Generalized and focal	15q	TIII	Small ossification center with three focal defects	Yes	Incomplete ossification and osteochondrosis
5	Focal	4g	CTB	Small bilobed defect	One lobe captured	Normal vascular dimple, see text
7	Focal	6r	CTB	Small bilobed defect with repair	Pale focus captured	Inconclusive, see text
12	Focal	10d	CTB	Small ovoid defect	Yes	Inconclusive, see text
		10b	CTB	Small ovoid defect	No	N/a
		10n	TIII	Small multi-lobulated defect	No	N/a
11	Focal	9e	CTB	Small multi-lobulated defect	No, but necrosis in articular cartilage	Early osteoarthritis
10	Focal	20j (kissing lesion with 20i)	TIII	Two medium to small cysts	Yes	Osteochondrosis, see Table 3
		20i	CTB	Medium cyst	No	N/a
6	Focal	16s (partly kissing with 16t)	CTB	Two large cylindrical defects	Yes	Osteochondrosis, see Table 3
		16t	TIII	Three large cylindrical defects	No, but osteo-chondrosis latens	N/a
13	Focal	22k (kissing lesion with 22l)	CTB	Medium cyst	Yes	Osteochondrosis, see Table 3
		22l	TIII	Large cyst	No	N/a
		22m	CTB	Small multi-lobulated defect	No	N/a
		22w (connecting stalk with 22x)	TIII	Large dorso-lateral shelf defect	Yes	Osteochondrosis, see Table 3
		22x	TIII	Large dorso-lateral groove in distal aspect	Yes	Osteochondrosis, see Table 3

[a]Numbers and letters refer to the lesion identification numbers in Sigurdsson SF, Olstad K, Ley CJ, Bjornsdottir S, Griffiths DJ, Fjordbakk CT. Radiological, vascular osteochondrosis occurs in the distal tarsus, and may cause osteoarthritis. Equine Vet J. 2022;54: 82-96. Abbreviations: CT, computed tomography. CTB, central tarsal bone. TIII, third tarsal bone.

Supplemental Table S6. Osteochondrosis lesions and secondary responses, in approximate order of size, severity and responses

			Primary osteochondrosis lesion		Secondary responses in cartilage		Secondary responses in bone			
**Case**	**Micro-CT lesion[a]**	**Bone**	**Cartilage canal necrosis**	**Chondrocytes surrounding necrotic canals**	**Adjacent chondrocyte proliferation**	**Adjacent canal proliferation**	**Delayed ossification**	**Fibro-vascular granulation tissue**	**Chondroclasts, osteoblasts**	**Diagnosis**
13	22k, medium cyst	CTB	Yes, eosinophilic streaks adjacent to and between lobes	Bilobed area of ischemic chondronecrosis in growth cartilage and granulation tissue in bone	Yes	No, adjacent cartilage avascular	Yes	Yes, shallow band	Chondroclasts	Osteochondrosis latens and pseudocyst (granulation tissue)
10	20j, two small to medium cysts	TIII	Yes, eosinophilic streaks	Low numbers of necrotic chondrocytes at margin of lesion	Yes	No, adjacent cartilage avascular	Yes	Yes, majority of defect and mostly solid	A few chondroclasts	Pseudocyst (granulation tissue)
6	16s, two cylindrical defects	CTB	Yes, eosinophilic streaks and necrotic canals, also inflamed patent canals	Areas of ischemic chondronecrosis and retained, viable, often hypertrophic chondrocytes	No	Yes, including osteoid-like matrix, centers of reparative ossification forming	Yes	No	Osteoblasts advancing into lesion lacunae	Hybrid articular and physeal osteochondrosis
6	16t, three cylindrical defects	TIII	Yes; necrotic canals, also inflamed patent canals	Area of ischemic chondronecrosis	No	No	No	No	No	Osteochondrosis latens
13	22x, distal groove defect	TIII	Yes, eosinophilic streak	Low numbers of necrotic chondrocytes	Yes	No, adjacent cartilage avascular	Yes	Yes; very small areas	Chondroclasts	Osteochondrosis manifesta
13	22w, dorsal shelf defect	TIII	Yes, can follow necrotic vessel into 22x	Areas of ischemic chondronecrosis	No	Not applicable: no adjacent cartilage or canals	Yes	Yes, majority of defect	Reparative ossification of granulation tissue	Osteochondrosis manifesta (granulation tissue)

[a]Numbers and letters refer to the lesion identification numbers in Sigurdsson SF, Olstad K, Ley CJ, Bjornsdottir S, Griffiths DJ, Fjordbakk CT. Radiological, vascular osteochondrosis occurs in the distal tarsus, and may cause osteoarthritis. Equine Vet J. 2022;54: 82-96. Abbreviations: CT, computed tomography. CTB: central tarsal bone, TIII: third tarsal bone.
